# Magnesium and Anti-phosphate Treatment with Bisphosphonates for Generalised Arterial Calcification of Infancy: A Case Report

**DOI:** 10.4274/jcrpe.galenos.2018.2018.0204

**Published:** 2019-09-03

**Authors:** Fatma Dursun, Tülay Atasoy Öztürk, Serçin Güven, Heves Kırmızıbekmez, Gülcan Seymen Karabulut, Sevinç Kalın, Betül Sözeri

**Affiliations:** 1 Ümraniye Training and Research Hospital, Clinic of Pediatric Endocrinology, İstanbul, Turkey; 2 Ümraniye Training and Research Hospital, Clinic of Radiology, İstanbul, Turkey; 3 Ümraniye Training and Research Hospital, Clinic of Pediatric Nephrology, İstanbul, Turkey; 4 Ümraniye Training and Research Hospital, Clinic of Pediatric Rheumatology, İstanbul, Turkey

**Keywords:** Generalized arterial calcification, infant, treatment, magnesium, etidronate

## Abstract

Generalized arterial calcification of infancy (GACI) is a rare autosomal-recessive disorder, characterized by calcification of the internal elastic lamina, fibrotic myointimal proliferation of muscular arteries and resultant arterial stenosis. Treatment with bisphosphonates has been proposed as a means of reducing arterial calcifications in GACI patients, although there is no formalized treatment approach. The case reported here was a patient with severe GACI diagnosed at three months of age who had no response to bisphosphonate treatment, but clinically improved after the initiation of magnesium and anti-phosphate (using calcium carbonate) treatments. In patients unresponsive to bisphosphonate, magnesium and anti-phosphate treatment may be attempted.

**What is already known on this topic?**

Generalized arterial calcification of infancy (GACI) is a rare disease that is associated with a high mortality rate owing to the development of severe hypertension and cardiovascular complications. GACI may begin *in utero* during the third trimester and 50% of children with GACI present with well-developed large arterial calcifications within the first week of life. Although there is no definitive treatment, it is claimed that patients treated with bisphosphonates have better survival rates. In contrast, children not treated with bisphosphonates were also reported to have spontaneous regression of large arterial calcifications. Furthermore, magnesium treatment has been reported to be beneficial in some experimental animal models.

**What this study adds?**

To date, only a few experimental treatment modes, other than bisphosphonates, have been proposed for GACI patients. This is a report of a patient who did not respond to bisphosphonates alone, but who subsequently improved clinically after treatment with magnesium and anti-phosphate therapy (calcium carbonate) along with continued bisphosphonates therapy.

## Introduction

Generalized arterial calcification of infancy (GACI) is a rare autosomal-recessive disorder, characterized by calcification of the internal elastic lamina, fibrotic myointimal proliferation of muscular arteries and resultant arterial stenosis (1,2). An extravascular feature is that foci of periarticular calcification occur in many of the affected subjects. Depending on the severity and the local distribution of the calcific stenoses, affected patients can present with neonatal heart failure, arterial hypertension and death within the first six months of life (3,4).

GACI is linked to mutations in the ectonucleotide pyrophosphatase/phosphodiesterase 1 *(ENPP1)* gene, which encodes for ectonucleotide pyrophosphatase/phosphodiesterase 1 *(ENPP1)*. This enzyme facilitates hydrolysis of adenosine triphosphate to adenosine 5’-phosphate and inorganic pyrophosphate (PPi). PPi is a potent inhibitor of hydroxyapatite crystal deposition, while inorganic phosphate (Pi) serves as a pro-mineralization factor. Thus an appropriate ratio of PPi/Pi is required to prevent spontaneous calcium phosphate precipitation. In patients with GACI, deficiency of the ENPP1 enzyme leads to reduced PPi/Pi and ectopic mineralization (5,6,7). In addition, *ENNP1* gene mutations have been identified in some patients with pseudoxanthoma elasticum (PXE), another hereditary ectopic mineralization disorder. Most cases with PXE also harbor mutations in the *ABCC6* gene (8). Recent studies have demonstrated a considerable genotypic and phenotypic overlap between PXE and GACI (9).

There is no effective and formalized treatment approach for patients affected by GACI (6). After the original report by Meradji et al (10), first-generation bisphosphonates, which are synthetic analogues of PPi, have been widely used in an attempt to treat GACI patients. First-generation bisphosphanates have a stronger effect in inhibiting formation and further growth of hydroxyapatite crystals compared to newer generation bisphosphonates (5,11). However, a potential complication of bisphosphonates is severe skeletal toxicity associated with prolonged use in patients with GACI (6). In addition to the skeletal toxicity, bisphosphonate treated children were also reported to experience persistent calcifications, which is an unwanted side effect of the treatment. Furthermore, some children not treated with bisphosphonates were reported to have spontaneous regression of large arterial calcifications (12,13,14). The lack of consistency and limited data concerning the efficacy of these compounds has created difficulties in reaching a consensus on the safety and efficacy of bisphosphonate treatment for GACI.

Li et al (15) investigated the dual effects of bisphosphonates on ectopic skin and vascular soft tissue mineralization versus bone microarchitecture in a mouse model of GACI. Their results suggested that bisphosphonate treatment may be beneficial for preventing ectopic soft tissue mineralization while correcting decreased bone mineralization. Effects of etidronate and alendronate on ectopic calcifications in the *ENPP1asj* mice were assessed at three different concentrations; doses the same as or five or 12 times greater than those used for treatment of osteoporosis. It was found that five times and 12 times greater doses of etidronate provided some benefit for reducing calcifications of the kidney, heart, descending thoracic aorta, or the eye.

Albright et al (16) used the identical animal model to evaluate the efficacy of ENPP1 enzyme replacement therapy in GACI. In this study, the breeding pairs were placed on the ‘acceleration diet’ to mimic the *in utero *calcification induced by ENPP1 deficiency and death was used as a preclinical endpoint. The results using ENPP1-enzyme replacement in this more severe preclinical study was a complete suppression of all ectopic calcification, as well as elimination of mortality. This study suggested that the efficacy of bisphosphonates was quite limited compared with what can be achieved with other more rational therapeutic interventions.

In a recent clinical trial of patients with PXE, the possibility of supplementing the diet with magnesium as a way of preventing mineralization was investigated (17). Kingman et al (8) showed the effects of dietary magnesium supplementation on ectopic mineralization in the vascular tissues in mice, a model for GACI, which shares genotypic and phenotypic overlap with PXE. Furthermore Rutsch et al (4) reported that application of a phosphate poor diet or a phosphate binding agent would be of interest with respect to early intervention in GACI. However, because this was a retrospective, small sized study with 55 subjects, it was difficult to draw any definite conclusion that patients with GACI may benefit from anti-phosphate treatment consisting of calcium carbonate supplementation.

In this case report, we report a case of a 3-month-old boy diagnosed with severe GACI who was unresponsive to bisphosphonate therapy but recovered after magnesium and calcium carbonate treatment in conjunction with continued bisphosphonate therapy.

## Case Report

A three month old male infant with para-articular calcification was referred to the paediatric endocrinology department of our hospital. The patient’s history revealed referral to the neonatology clinic at age 17 days because of arthritis in the right hip which had been noted in the first week of life. The infant was the second child of a 39-year old healthy mother and a 37-year old healthy father who were first degree cousins. He also had a three year-old healthy brother. The patient had been delivered by caesarean section at the gestational age of 38 weeks. Birthweight was 3680 g.

Septic arthritis was suspected, but acute phase reactants and cultures were negative. Histopathologic investigation of a biopsy specimen obtained from the right hip joint revealed severe calcification in the arterial walls with no evidence of inflammation.

**Figure 1:**
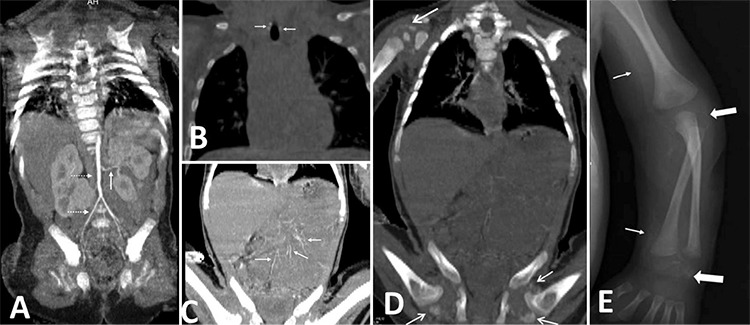
At age three months, coronal non-contrast computed tomography of abdomen and chest, A) Diffuse narrowing is seen in bilateral renal arteries, abdominal aorta and bilateral iliac arteries, B) At the level of the larynx, soft tissue calcifications are observed in the paratracheal region and around the hyoid bone, C) There are linear hyperdensities consistent with calcification in the mesenteric artery and branches, D) Periarticular calcification in the right shoulder joint and in the right hip joint, E) At age three months, on baseline radiograph; the left wrist shows arterial calcification of the brachial and radial arteries (thin arrow) and intra- and peri-articular calcifications in left elbow and wrist joints (thick arrow)

At presentation, the patient’s weight was 4900 g [-1.72 standard deviation (SD) score (SDS)] and his length was 58 cm (-1.22 SDS). He had prominent ears. Systemic physical examination was normal except for a swollen, painful and restricted right hip joint. Arterial blood pressure was measured at 121/84 mmHg, which was high (>95^th^ percentile) for a three month old boy. Echocardiography showed a normal left ventricle wall and coronary artery thickness. Audiologic and ophthalmologic assessments were normal. Routine biochemical tests were normal while plasma renin activity and aldosterone levels were above normal reference ranges (Table 1). Non-contrast abdominal computed tomography (CT) was performed. Diffuse narrowing of the abdominal aorta, bilateral renal arteries and iliac arteries was observed (Figure 1A). Soft tissue calcifications were observed in the paratracheal region at the laryngeal level and around the hyoid bone (Figure 1B). There were linear hyperdensities, consistent with calcification, in the mesenteric artery and its branches (Figure 1C). Periarticular calcifications in the right shoulder and right hip were observed (Figure 1D). Baseline radiographic images revealed arterial calcifications in the brachial and radial arteries on the left side and intra- and peri-articular calcifications in the left elbow and wrist joints (Figure 1E). There was no evidence of calcification in the cerebral arterial vessels on cranial CT. Due to the severe arterial calcification noted in the histopathologic investigation, a diagnosis of GACI was considered and *ENPP1* gene analysis was performed. A previously identified homozygote (c.2677G>T p.E893*) (p.Glu893*) mutation was detected in the *ENPP1* gene. The genetic analyses of the parents was not performed since the mutation was a previously reported one; however they have received genetic counselling.

**Figure 2:**
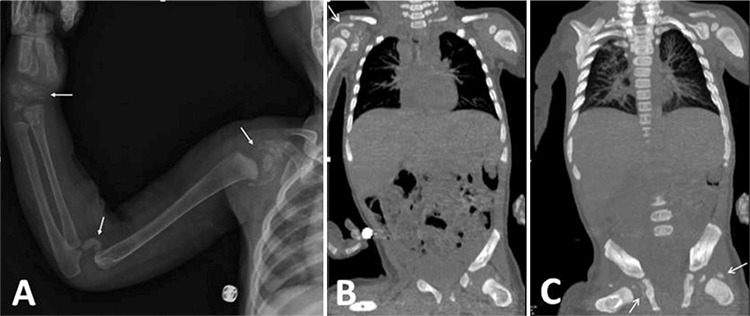
At age nine months, radiograph before magnesium and anti-phosphate treatment, A) Radiograph shows progression of periarticular calcification in the right shoulder, elbow and wrist joint, B) Coronal non-contrast computed tomography of abdomen demonstrates periarticular calcification in the right shoulder joint, and C) shows periarticular calcification in right hip joint

Intravenous disodium pamidronate was administered as three doses on days 0, 7 and 10. On the fifth day of pamidronate treatment, oral etidronate was initiated at a dose of 10 mg/kg/day which was increased to 20 mg/kg/day after three days. After six months of etidronate treatment, calcifications on direct radiographs and CT persisted (Figure 2A, 2B, 2C) as well as intermittent swelling and restriction of joints. This suggested an inadequate response to biphosphonate treatment. Calcium carbonate treatment at a dose of 250 mg twice a day and magnesium oxide treatment 150 mg twice a day were started with a simultaneous reduction in Etidronate to a dose of 10 mg/kg/day. While calcium, phosphorus and other laboratory parameters were normal at baseline (Table 1), serum phosphorus concentration decreased following the anti-phosphate treatment, as expected. After the initiation of calcium carbonate and magnesium treatment, restriction and swelling of the joints gradually improved. No adverse effects were experienced in the follow-up period. A marked decrease of calcifications was seen in the radiographs which were taken during the sixth month of treatment. Calcium carbonate and magnesium treatments were continued while etidronate was further reduced to a dose of 5 mg/kg/day.

**Figure 3:**
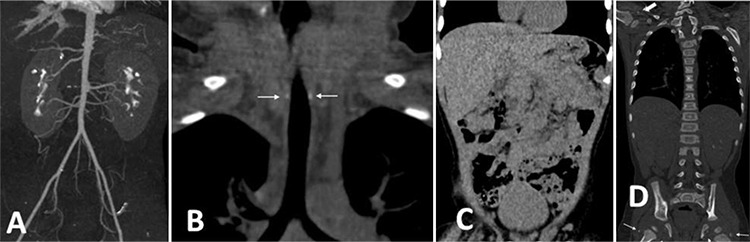
Computed tomography (CT) angiography images and non-contrast CT of abdomen and chest after treatment with magnesium demonstrates: A) Normal appearance of abdominal aorta, bilateral internal and external iliac arteries, femoral artery, renal and mesenteric arteries, B) At the level of the larynx, soft tissue calcifications are reduced in the paratracheal region and around the hyoid bone. C) Mesenteric artery wall calcifications are not observed. D) Periarticular calcification in the right shoulder joint and in the right hip joint are reduced

**Figure 4:**
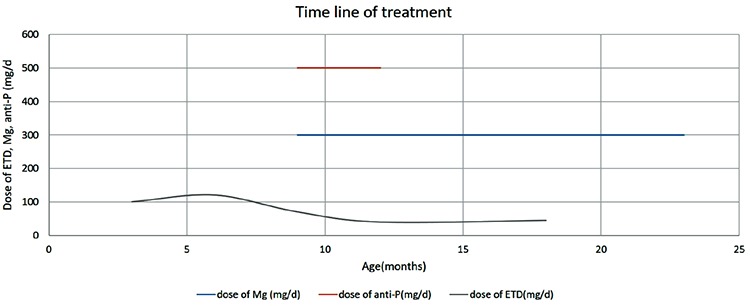
Time line chart of treatments received by the patient Mg: magnesium, ETD: etidronate, anti-P: anti-phosphate

CT and CT angiography were performed at the end of the first year of calcium carbonate and magnesium treatments. The calcifications previously observed in the abdominal and mesenteric arteries had disappeared, there was no longer any narrowing of renal arteries evident and there was a significant reduction in calcifications in hip and shoulder joints (Figure 3A, 3B, 3C, 3D). In addition there was a significant clinical improvement in joint functions and motor development. At the most recent examination of the patient, at the age of 23 months, his weight was 10 kg (-1.93 SD), height was 85 cm (-0.7 SD), arterial blood pressure measurements were normal, joint movements were comfortable and neuromotor development was improving. The etidronate treatment was stopped and magnesium treatment was continued. The course of treatment is shown in Figure 4.

Informed consent was obtained from the parents of the patient for publication of this case.

## Discussion

Our patient presented with arthritis at the age of three months. He was diagnosed as GACI and treated with etidronate, magnesium and anti-phosphate. Approximately 50% of children with GACI present within the first week of life with large arterial calcifications which are reported to develop as early as the third trimester of pregnancy. The course of these children may be less favorable than children who present later (4). Although our patient was diagnosed with GACI at three months old, clinical findings consistent with GACI were reported to have been present during the first week of life.

Respiratory distress is one of the presenting features in more than 50% of cases, followed by feeding intolerance, poor weight gain, tachypnea, tachycardia and cyanosis (4,18,19). The disease usually results in death in infancy due to progressive ischemic heart failure associated with coronary calcification. Survivors of GACI frequently present with periarticular calcifications rather than coronary calcification (4).

Treatment options in GACI are limited to the use of bisphosphonates, such as etidronate and pamidronate (18). Bisphosphonates are synthetic analogs of inorganic pyrophosphate, which block the conversion of calcium phosphate to hydroxyapatite and thus may reduce ectopic calcification (19). Etidronate, as a first-generation bisphosphonate, has been used most frequently at a dose of 5-35 mg/kg/per day orally (5). Etidronate has a stronger effect in inhibiting mineralization compared to the newer aminobisphosphonates and shows no adverse effect on growth (19). However, high-dose etidronate injections have been shown to induce vitamin D-resistant rickets in rats (20). Other nitrogen-containing bisphosphonates, which have been used in earlier case series reports of GACI, include intravenous pamidronate and oral risedronate (5,21). *In vitro* studies have shown that bisphosphonates accumulate within vessel walls suggesting that these drugs may have a direct effect on calcification (6). As the starting treatment, we administered three intravenous doses of pamidronate infusion, in accordance with previous reports (6). Subsequently, oral etidronate was added to the treatment.

It is difficult to evaluate whether recovery occurs spontaneously or with the effect of bisphosphonates. Long-term survival has been reported in GACI patients with no specific therapy, thus the possibility of spontaneous resolution of calcification should be considered (12,14). In a retrospective study it was reported that 17 of 55 patients affected by GACI were treated with bisphosphonates, namely etidronate, pamidronate, clodronate or risedronate. Survival rate of these treated patients was found to be 65%, while 69% of patients who were not treated with bisphosphonates had died in infancy (4). These authors have also claimed that children treated with bisphosphonates have a survival advantage, but this claim was based on observations in a retrospective study with a small sample size rather than a blinded clinical trial. Indeed, the survival advantage suggested by these authors was not statistically significant. More favorable outcomes in some children could be related to disease severity. Patients with less severe disease may survive long enough to be transported to a medical center, evaluated and treated with bisphosphonates. The persistence of calcifications, especially in periarticular regions leading to severe restriction and contractures, indicated a need for exploration of alternative therapeutic options. Magnesium treatment was reported to be effective in *ENPP1* knockout mice. In a recent study, Kingman et al (8) showed that elevated dietary magnesium during pregnancy and postnatal life prevents ectopic mineralization in *ENPP1asj* mice, a model for GACI. Based on this experimental report, oral magnesium oxide treatment at a dose of 150 mg twice a day was commenced in our patient.

The mechanism for the inhibition of ectopic mineralization by magnesium may involve direct interactions between magnesium and calcium ions in the mineralization process. Magnesium competes with calcium, reduces calcium-phosphate binding and forms magnesium phosphate complexes. These complexes, which are soluble, prevent mineral deposition (8).

Phosphate levels are high in healthy newborns, probably due to low glomerular filtration rate and retention of phosphate (22). These higher levels may lead to an increased risk of arterial calcification in young patients with ENPP1 deficiency during the first few months of life, which may decline with age (6). PPi and Pi seem to have mutually antagonistic roles in tissue mineralization. Significantly, a phosphate-poor diet induces hypophosphatemia with markedly decreased artery calcification and periarticular calcifications. Furthermore, several mutations in the *ENPP1* gene result in the phenotype of autosomal recessive hypophosphatemic rickets (ARHR2) without any arterial calcifications (5,23). Furthermore in some patients with generalised arterial calcification due to *ENPP1* mutations in infancy, hypophosphatemic rickets developed in the following years. Treating these patients with calcitriol and phosphorus led to the recurrence of calcifications. It has been reported that hypophosphatemia is a protective factor against vascular calcification (24). Rutsch et al (4) found that both hypophosphatemia and hyperphosphaturia are associated with GACI survival. Both the hypophosphatemia and hyperphosphaturia were linked to increased fibroblast growth factor 23 (FGF23) concentrations. FGF23 is a hormone which induces phosphate wasting in the urine. GACI patients with elevated FGF23 and low phosphate are expected to have reduced vascular calcifications. Therefore, phosphate wasting via increased FGF23 production may be an adaptive mechanism in GACI to accommodate the low plasma PPi by reducing plasma Pi in an attempt to preserve the Pi/PPi ratio. A consequence of the hyperphosphaturia is osteomalacia and rickets, seen in ARHR2. This may indicate an association between ARHR2 and GACI (25). Since arterial calcifications could be lethal in infancy, thesepreviously reported observations encouraged the hypothesis that creating a controlled hypophosphatemia could decrease mortality. A careful and closely monitored balance between bone demineralisation and arterial calcifications should be sought. In our patient, treatment with both magnesium and calcium carbonate was started with the aim of lowering phosphate levels and keeping the magnesium levels within the upper limit. As shown in Table 1, there was a significant clinical improvement after the initiation of concurrent magnesium and calcium carbonate treatment. Joint mobility improved and hypertension recovered. Marked reduction in calcifications was detected on direct radiographs and CT performed at the after 12 months of this combined therapy. No adverse effects were observed during the treatment process.

The limitation of this study include a lack of adequate experience in treating patients with GACI and, since recovery of calcifications in GACI occur spontaneously in some of the survivors in the absence of any therapeutic intervention, it is difficult to conclude that the improvement was due to the treatment protocols. Due to inadequate knowledge of the natural history of GACI, we hesitated to cease the bisphosphonate treatment at the beginning of the magnesium and calcium carbonate treatment. Between the sixth and twelfth month of this additional therapy, the patient also received etidronate at gradually reduced doses, together with other treatments. Thus, in this case it is impossible to ascertain the relative benefits of the treatments given, including the effects of varying doses of etidronate, although the outcome at nearly two years of age appears clinically good.

In the current case, a sufficient clinical response was not obtained after six months of etidronate treatment. Bisphosphonate treatment had not made a significant impact on the regression of calcifications. Although there was insufficient information in the literature concerning magnesium and calcium carbonate treatment, there are animal data showing that these could be effective. A significant reduction in calcifications after the initiation of magnesium and calcium carbonate treatment was observed in our patient. Thus we believe that this treatment option should be considered in GACI patients, especially in those in whom bisphosphonates appear clinically ineffective. Further case reports and, ideally, carefully designed studies would help to resolve this matter in the future.

## Figures and Tables

**Table 1:**
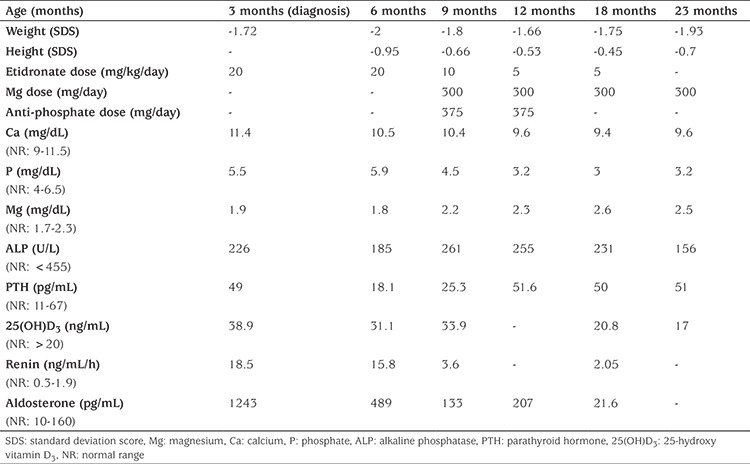
Laboratory and auxological findings of the patient under treatment
